# An emotional modulation model as signature for the identification of children developmental disorders

**DOI:** 10.1038/s41598-018-32454-7

**Published:** 2018-09-27

**Authors:** Arianna Mencattini, Francesco Mosciano, Maria Colomba Comes, Tania Di Gregorio, Grazia Raguso, Elena Daprati, Fabien Ringeval, Bjorn Schuller, Corrado Di Natale, Eugenio Martinelli

**Affiliations:** 1 Department of Electronic Engineering, University of Rome Tor Vergata, via del Politecnico 1, 00133 Roma, Italy; 2 Faculty of Science MM.FF.NN., University of Bari, Aldo Moro, University Campus Ernesto Quagliariello, Via Edoardo Orabona 4, 70126 Bari, Italy; 3 Department of Systems Medicine, CBMS, University of Rome Tor Vergata, via Montpellier 1, 00133 Roma, Italy; 4 Laboratoire d’Informatique de Grenoble, Université Grenoble Alpes, 38401 St Martin d’Hères, France; 5 GLAM – Group on Language, Audio & Music, Imperial College London, SW7 2AZ London, UK; 6 Chair of Embedded Intelligence for Health Care and Wellbeing, University of Augsburg, 86159 Augsburg, Germany

## Abstract

In recent years, applications like Apple’s Siri or Microsoft’s Cortana have created the illusion that one can actually “chat” with a machine. However, a perfectly natural human-machine interaction is far from real as none of these tools can empathize. This issue has raised an increasing interest in speech emotion recognition systems, as the possibility to detect the emotional state of the speaker. This possibility seems relevant to a broad number of domains, ranging from man-machine interfaces to those of diagnostics. With this in mind, in the present work, we explored the possibility of applying a precision approach to the development of a statistical learning algorithm aimed at classifying samples of speech produced by children with developmental disorders(DD) and typically developing(TD) children. Under the assumption that acoustic features of vocal production could not be efficiently used as a direct marker of DD, we propose to apply the Emotional Modulation function(EMF) concept, rather than running analyses on acoustic features per se to identify the different classes. The novel paradigm was applied to the French Child Pathological & Emotional Speech Database obtaining a final accuracy of 0.79, with maximum performance reached in recognizing language impairment (0.92) and autism disorder (0.82).

## Introduction

Star Trek fans will remember EMH, the Emergency Medical Holographic program that had the appearance of a reliable, middle aged family doctor. Even if we are miles away from developing an artificial healthcare practitioner, in recent years, significant advancements have been made in computer-aided diagnosis, digital technology and artificial-intelligence support to clinical practice^1–8^.

Promising opportunities come from the domain of machine learning and, specifically, from supervised^9^ and unsupervised^10^ learning machines. Such approaches typically require extraction of a set of features that characterize the items at study (e.g., colour, frequency, wavelength …) and involve a classification method capable of distinguishing and assigning items to separate classes. Through machine learning, algorithms can automatically extract features from the available data and implement classification by using a reference data set (*training set*) for which labelling is known. Once trained, the classifier works on its own, allowing for huge amounts of data to be rapidly labelled, an approach that has proved successful in a number of domains (e.g., diagnostic imaging, remote sensing imaging …).

In recent years, the interest for Big Data analysis has extended to the area of psychiatry research^5^, providing novel ways to classify brain disorders from abnormalities in neuroimaging and/or genomic data^11–14^ and introducing new methods to predict the outcome of therapeutic approaches^15^. The obvious advantage of integrating clinical practice with information drawn from statistical learning rests on the opportunity to speed up the entire diagnostic procedure, which – in turn – can reduce frustration, adverse outcomes and prolonged disability in patients. These benefits are especially relevant to the paediatric population since early diagnoses of neurodevelopmental disorders, such as Autism Spectrum Disorders (ASD) or Attention Deficit Hyper-Activity Disorder (ADHD), can promote timely intervention, positively influencing the children’s future lives.

When data from the clinical domain is concerned, a problem faced by most machine learning methods is the heterogeneity of presentation of most diseases. For example, in the case of ASD, highly heterogeneous patterns are described for genetic profiles^16^, gender-specific effects^17^ language phenotypes^18^ and more, to the point that it has often been suggested that autism should not be considered as a single disorder but rather as ‘*the autisms*’^19^ – hence the term *spectrum* in ASD. In terms of machine learning applications, heterogeneity can hinder efficiency of classifiers, making predictions less reliable. Whereas some recent approaches exploit generative adversarial networks to augment artificially the data space^20^, other methods can be drawn from the fast-developing area of ‘*personalized medicine*’, i.e. the growing knowledge that diagnostic and therapeutic strategies should take variability into account, thence applying highly individualized approaches to patients. This message – that has been largely received in the domain of oncology^21^ – is becoming increasingly more relevant also to studies in other areas, including neuropsychiatry (see for instance^22–24^).

**Figure 1:**
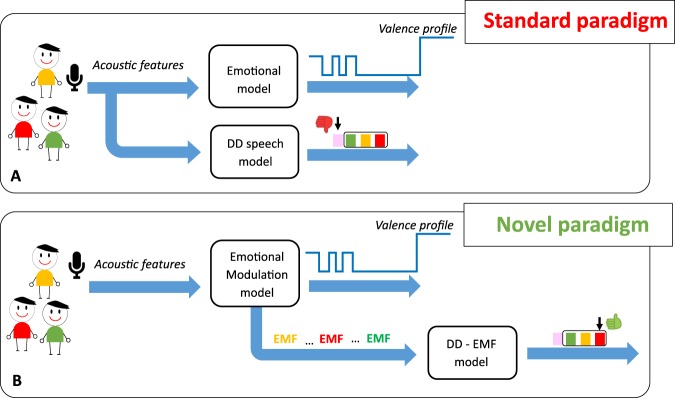
Comparison of standard and novel paradigm. Panel A. Acoustic features extracted from the recorded speech are used to recognize the expressed emotion (Emotional model) (e.g., valence profile) and the pathology (DD speech model). Panel B. Acoustic features are used to construct the emotional modulation model. The Emotional Modulation Function (EMF) of different subjects is then used to train an DD – EMF model.

Stemming from this idea, we explored the possibility of applying an emotion-driven approach to the development of a personalized statistical learning algorithm aimed at classifying samples of speeches produced by typically developing children (TD) and by children with autism disorder (AD), specific language impairment (SLI), Pervasive Developmental Disorder-Not Otherwise Specified (PDD-NOS). The latter three conditions are characterized - to different degrees - by severe deficits in social interactions and communication skills, as well as by stereotyped behaviors^25^. In addition, when speech is concerned, all three conditions are known to induce a flat, monotone intonation and anomalies in the use of volume, pitch and stress^26–32^. Accordingly, for the purpose of this investigation, children with AD, SLI, and PDD-NOS will be considered as a single group, broadly labeled here as Developmental Disorders (DD)^32^. We used acoustic features to automatically differentiate children with DD from children with TD diagnosis using speech recordings^2,33^. A recent meta-analysis^34^ has pointed out that acoustic features of vocal production can indeed be used as a marker of ASD, even if the over 30 papers reviewed failed to identify a single characterizing feature. Recently, a study has described a machine learning strategy that recognizes spontaneous emotional expressions in the voice and discriminates DD individuals from TD children based on speech features^35^. However, the proposed method did not account for the variability in emotional expression within individuals although this aspect can impact on characterization of the pathology. To counteract this limitation, we introduce a novel signal processing paradigm that exploits the individual emotional modulation occurring during speech in order to model atypical behaviors that are symptomatic of DD. The present paradigm thus departs from traditional approaches that directly learn acoustic models from the speech signal of DD children, while treating the corresponding valence profile in parallel. For a better outline of the novel paradigm, Fig. 1 compares the standard and the novel paradigm.

In the standard paradigm (Panel A), the acoustic features extracted from the recorded speech are used to construct the emotional model that estimates a valence profile of an individual^34,35^. The same features are then used to train the DD speech model. Pink, green, yellow and red boxes represent the different subcategories of children (TD, PDD-NOS, SLI, and AD respectively). The limits of this approach will be outlined in section 2.2. In the novel paradigm (Panel B), the acoustic features are used to construct the emotional model as before, if needed. However, a different concept, the Emotional Modulation function (EMF) represented by the regression coefficient of the emotional model, is used to train a model of the pathology. The EMF represents the way each individual modulates his/her emotional response to a given known stimulus. More specifically, EMF is the leading concept in our rationale that fosters new scenario in understanding atypical behaviors that are symptomatic of DD. Numerically, EMF quantifies the *weights* that each individual spontaneously gives to his/her voice alterations in order to encode non-verbal information in speech. The weights are the core of the new way of thinking since we believe their dynamic is relevant to the different DD manifestation and can be then used to learn an appropriate representation. Most importantly, this novel approach ensures the possibility to construct a personalized model of emotion first, and successively to use the associated EMF to predict the class to which the child belongs (e.g. AD, PDD-NOS, SLI, or TD).

In the present work, we will apply the novel paradigm to the French Child Pathological & Emotional Speech Database (CPESD)^35^. For compilation of the database, children aged 6–18 were involved in an unconstrained task: a story telling of a pictured book^36^. It was assumed that the children’s production of prosodic cues during the telling of the story was correlated to the level of emotional valence elicited by each picture of the book, which was assessed in three categories by a psychologist (negative, neutral and positive). The dataset includes 102 individuals reported respectively as DD (N = 34) and TD (N = 68), with a ratio of two TD for one DD child of same age and sex. Acoustic features contained in each utterance produced by children during the story telling were then extracted for further model development.

## Results

In order to evaluate the performance obtained by the proposed methodology, we ran three different tests. The first presents the personalized model of emotion constructed for each participant, independently from the corresponding diagnosis. The second provides evidence for the general failure of the standard paradigm (Panel A Fig. 1), as shown by the computation of the balanced accuracy (ACC) on the children’s groups when acoustic descriptors are solely used to construct the recognition system; ACC is an evaluation metric that compensates uneven class distribution by computing the average recall per class. Finally, as a third test, we present the performance of classification when the novel paradigm (DD-EMF model) is applied in recognizing TD from DD subjects. In the dataset, children with a diagnosis of a disorder belonged to one of the three following categories: AD (N = 11), PDD-NOS (N = 13) or SLI (N = 10), which, for the sake of simplicity, will henceforth be all labelled comprehensively as DD. For the third test, the low number of participants in each subcategory does not justify development of a four-classes recognition problem. Therefore, we investigated the two-class problem of recognizing typical vs atypical children from their voice (i.e., TD vs DD).

### Test 1. Performance of the valence recognition model

**Figure 2:**
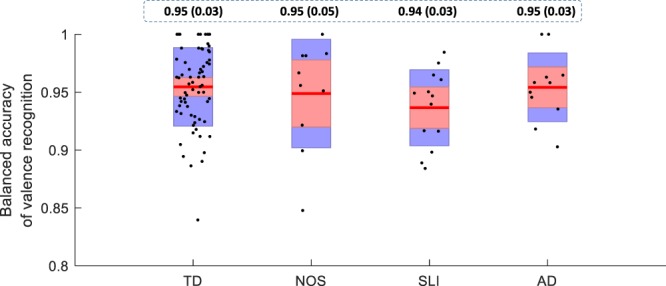
Average balanced accuracy of valence level recognition. Boxplots of the ACC representing the valence level recognition performance on three classes (chance score is 0.33). From left to right: TD, PDD-NOS, SLI, and AD subjects. Dots represent the different participants, central pink area represents the interquartile range [25th–75th] percentiles and the blue dashed area represent the [10th–90th] percentiles range. Values in the dashed board represent average (standard deviation) values.

To demonstrate the appropriateness of the acoustic features in describing the emotional valence in an individual’s speech sample, we constructed a three-class classifier based on Linear Discriminant Analysis (LDA). The three classes, labelled as −1, 0, and 1, represent negative, neutral, and positive valence, as codified in the dataset. Features are preliminarily selected using thresholding of the individual Pearson’s correlation coefficient (ρ_c_) with respect to the valence level assessed for a given subject. Only features with an absolute ρ_c_ value larger than 0.7 in the training dataset will be kept and used for further analysis. The accuracy of the three classes, computed using a leave-one-utterance-out cross-validation procedure, is estimated separately for each disorder and for the TD subjects and collected in the boxplots shown in Fig. 2. It can be observed that accuracy is around 0.95 in all categories of children indicating a very strong effectiveness of the selected features in modelling the emotional valence, independently from the presence of DD disorder. On the other hand, this is an indirect demonstration of the fact that acoustic features equally behave with respect to the presence of disorder.

### Low performances of the standard paradigm in DD recognition

**Figure 3:**
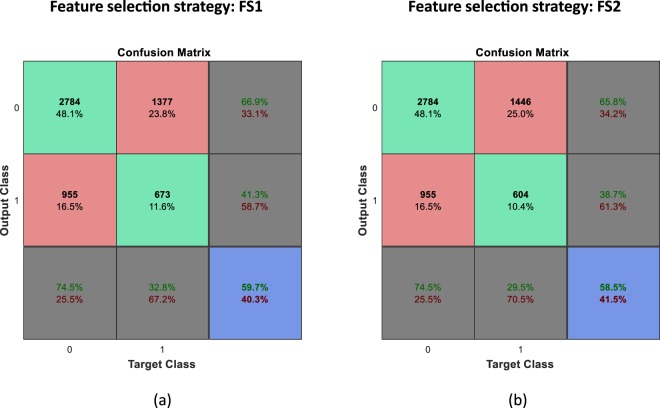
Confusion matrix in the standard paradigm. Confusion matrix obtained (**a**) using an emotional-driven feature selection strategy (FS1) and Support Vector Machine (SVM) classification, and (**b**) implementing a diagnosis-based feature selection approach (FS2) in the training set and Support Vector Machine (SVM) classification. A blue box represents the overall accuracy of the results (green boxes = percentage of success and red boxes = percentage of failure).

To highlight the importance of the novel paradigm for DD recognition, we first provide results obtained by using the standard approach (Fig. 1 Panel A). In particular, we consider two different model settings (henceforth described as FS1 and FS2). In the first (FS1), the binary classification model for discrimination of DD from TD individuals was trained directly on the acoustic features extracted from each utterance. In this scenario, each utterance is a row and each acoustic feature is a column of the data matrix. Features were selected by implementing a two-sided Student t-test ranking criterion with respect to the diagnosis collected in the training partition and the first 100 ranked features were retained to construct the classification model. In the second test (FS2), the binary classification model was trained on the features selected according to the maximum correlation with the valence annotation of the corresponding subject in the training partition. The rationale behind these tests is to present a way to improve the standard paradigm regarding the direct recognition of DD through the child’s vocal expression. A Support Vector Machine (SVM) with a linear kernel and default parameters setting (box-constraint parameter value set to one and feature weighted standardization) has been implemented in both tests to assign the final label of TD or DD to each utterance. Figure 3 shows the confusion matrices obtained in the two cases.

Results clearly show that in both situations, the average recognition rate is unacceptably low (59.7% and 58.5% respectively), and hence discredits the assumption of a direct relationship between acoustic features and the child’s diagnosis.

### Performance of the novel DD recognition paradigm

**Figure 4:**
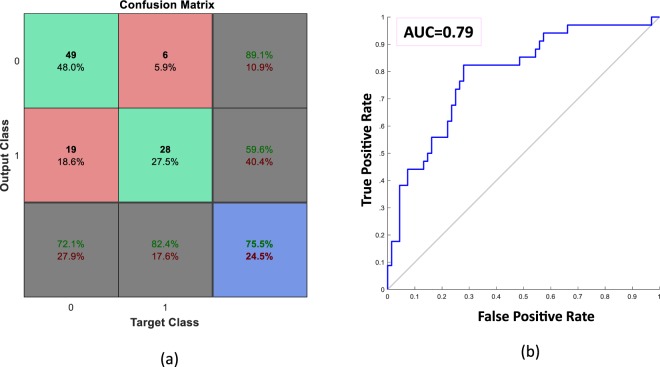
Results of the proposed EMF paradigm. (**a**) Confusion matrix of the recognition performance obtained by the proposed approach. Grey boxes at the bottom indicate the average recognition rate of TD subjects (72.1% Specificity) and of subjects with DD (82.4% Sensitivity or Recall), leading to an unbalanced accuracy equal to 75.5% (blue box). Balanced accuracy ACC is equal to 77%. Positive Predictive Value (PPV) is equal to 59.6% and false omission rate (FOR) is equal to 89.1%. (**b**) The Area under the Receiving operating curve (AUC) value equals to 0.79.

The novel approach stems from the assumption that the way the child modulates his/her emotional speech towards a given emotional stimulus could be characteristic for the subjects suffering from DD. One risk with this approach is the high heterogeneity in the children’s capacity to verbally express their emotional attitude. To overcome this problem, we begin by computing average descriptors of all the utterances by the same subject. To do this, after having collected features that mostly correlate with the valence level (see Section 3.3), we combine the values of such descriptors over all the utterances assessed with the same valence level for one subject (all utterances assigned a negative valence, all with assigned neutral valence and all assessed with positive valence levels, respectively). This step is motivated by the underlying assumption that not all of a child’s utterances could be considered as correlated with a DD diagnosis. Moreover, this strategy allows us to combine verbal productions from different individuals, regardless of the speech length. Biasing in valence subjectivity of each participant is reduced by the expert-based assessment of the valence level performed by psychologist. By combining the information extracted from all the utterances for the same individual, this novel method further compensates for the unavoidable inaccuracy of this task. In addition, this combination is performed by using distribution descriptors such as the mean value, skewness and kurtosis computed over the utterances of the same individual labelled with the same valence category (e.g., all the utterances of an individual labelled with positive valence). The last two parameters (skewness and kurtosis) allow us to add to the average value of the feature over different utterances, the dispersion of the feature over the utterances of the same valence level. Recalling again that only 19 acoustic feature were extracted at the first step, then 57 high-level descriptors resulted (19 average values, 19 skewness values, and 19 kurtosis values). These descriptors are fed into a Multilinear Regression Block (MLR) block (see Section 3.3) with expected output valence category −1, 0 and 1. The corresponding 57 MLR coefficients are estimated and used. Such coefficients represent the EMF through which each child reacts to the administered emotional stimulus, and that, in our rationale, is expected to carry on the disorder symptoms (if any). The procedure is performed over each individual. Acoustic feature were selected using valence annotations and no other information regarding diagnosis. The DD model is indeed constructed using a supervised learning approach ran over different individuals, thus requiring a cross-validation procedure for training and validation. In particular, by implementing a leave-one-patient-out cross validation procedure, we ran the dynamic feature selection (DFS) approach (see Section 3.3) to dynamically select the features according to each test data^37,38^. A binary classification model based on an SVM, with linear kernel and default parameters setting, is then trained on the EMF matrix of all the subjects except for one that, in turn, is left out for test. The ACC is computed along the area under the Receiving Operating Characteristic (ROC) curve (i.e., AUC). Figure 4(a) shows the confusion matrix obtained in our test while Fig. 4(b) reports the ROC curve, with the corresponding AUC indicated.

### EFM coefficient and disorder’s diagnosis

**Figure 5:**
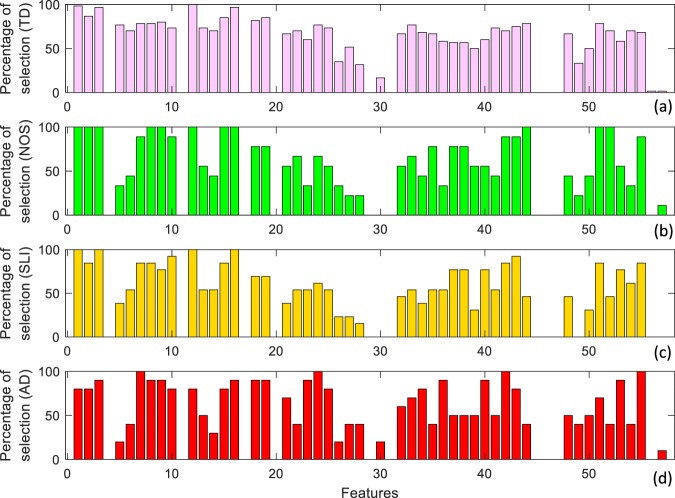
Percentage of feature selected. Percentage of feature selection for each of the 57 features obtained using the DFS approach (DD vs TD recognition) and displayed according to diagnosis. (**a**) TD children, (**b**) NOS children, (**c**) SLI children, (**d**) AD children. It is interesting to note that a very homogeneous group of features were selected for TD subjects (**a**) and similarly, selected features were almost the same across the three different groups of DD (**b**–**d**). This evidence reinforces the assumption that the value of the EFM coefficients can represent the different facets of the disorder.

Figure 5 describes the percentage of selection for each of the 57 features obtained using the DFS approach (DD vs TD recognition) and displayed according to diagnosis. Recall that the 57 features have been extracted by computing standard high-level statistical descriptors from 19 acoustic features. The 19 selected features belong to the well-defined groups described in Table 1^39^. All retained features are related to spectral and cepstral acoustic descriptors.

**Table 1:** Features group according to the categorization defined in^39^.

Feature N.	Features Group
1–16	RelAtive Spectral TrAnsform Perceptual Linear Prediction (RASTA-PLP) that considers temporal properties of the human hearing and speech production systems.
17	Functionals computed over spectral moments.
18–19	Functionals computed over Mel-Frequency Cepstral Coeffi cient (MFCC).

As can be seen from Fig. 5, on average, a very homogeneous group of features were selected for TD subjects (Fig. 5a). Similarly, selected features were almost the same across the three different groups of DD (Fig. 5b–d), thus reinforcing the assumption that the EFM coefficients can represent the different facets of the disorder. Moreover, due to the DFS mechanism, features were selected according to test data. Hence, features were differently chosen in presence of different diagnosis. For example, note that feature 30 has been selected only in presence of AD or TD, while feature 57 is totally absent in SLI and TD. Most relevantly, selection of features 20–28 (skewness of features RAST-PLP computed over utterances with equal valence) in AD subjects manifests a strong deviation from that of TD subjects, indicating a high specific behaviour with respect to the diagnosis.

**Figure 6:**
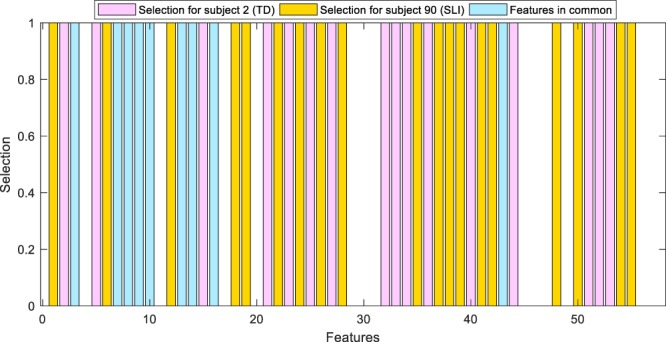
Two examples of feature selection. Pink bars denote features selected for a TD subject, yellow bars identify features selected for a SLI subject, whereas cyan bars locates features selected for both.

As an example, in Fig. 6, we further show the features selected for a TD subject compared with those selected for an SLI subject. Pink bars denote features selected for a TD subject (subject n. 2), yellow bars identify features selected for an SLI subject (subject n. 90), whereas cyan bars locate features selected for both. The limited number of cyan bars underlines the evident difference in features selected for the two subjects thus confirming the usefulness of the DFS approach.

Finally, in support to the efficiency of the novel paradigm, Table 2 lists the percentage of individuals from the different pathological subgroups (PDD-NOS, SLI, and AD) that were correctly included in the DD class.

**Table 2:** Rate of correctly classified individuals in each subcategory of DD: PDD-NOS, SLI, and AD.

	PDD-NOS	SLI	AD
Rate of classification	7/10 (70%)	12/13 (92%)	9/11 (82%)

TOT quantifies the total number of cases for each subcategory.

## Discussion

Experienced psychologists and psychiatrists can easily remark whether a subtle change in a patient’s voice betrays a change of mood or suggest a specific disorder. Present machine learning algorithms are not so good. Indeed, the experimental results described in Section 2.2 pose a dilemma to the domain of speech analysis: can acoustic features be used directly for classification of psychiatric disorders? The standard paradigm presents strong criticisms that have been previously reported in^35^ and are further demonstrated by the present test (Section 2.2), suggesting that the answer to the question is more likely ‘no’. The novel paradigm described here opens a promising alternative, indicating that by applying the Emotional Modulation function (EMF), rather than running analyses on acoustic features *per se*, the answer to the question could be turned to ‘yes’. The EMF is a quantitative way to model the emotional speech reaction of a single individual to a given stimulus. The rationale behind this method is that differently from acoustic features, the EMF ‘keeps’ the disorder’s traits. This specificity arises from the fact that EMF is a personalized concept, different for each individual. It encompasses the natural heterogeneity that individuals manifest during storytelling, verbal responding, and speech production in general.

As shown in Fig. 4, this novel approach allows to clearly separate traits that belong to TD individuals from those that characterize DD individuals. Moreover, although here, we only explored the binary classification problem of recognizing TD vs DD individuals, Table 1 clearly shows an excellent performance in the number of true positive subjects (TPs) included in each subcategory of DD. It is interesting to note that based on the approach used here, the PDD-NOS category is the class most likely to be confused with TD children. PDD-NOS corresponds to Pervasive Developmental Disorder-Not Otherwise Specified (PDD-NOS)^35^, which is characterised by social, communicative and/or stereotypic impairments that are less severe than in AD and appear later in life. Hence, it describes individuals likely to display more common features with TD participants. On the contrary, SLI impairment appears as the most evident disorder category, since Selective Language Impairment directly impacts on speech, and hence on the EMF. AD diagnosis reaches a recognition rate equal to 82%. Taken together, these results encourage research in this direction, with the acquisition of a larger dataset of children, thus, allowing the development of a 4-class recognition model.

By modelling the emotional speech reaction of each participant individually, the present method overcomes a frequent limitation of statistical approaches to clinical samples, i.e. their generalizability. ASD is a profoundly heterogeneous condition, meaning that sampling methods are likely to impact on the models that are developed. For example, a meta-analysis on studies on emotion recognition in ASD recently pointed out that females and individuals outside the typical IQ range are poorly represented in the common study populations^40^. Similarly, among the examined studies, half had been conducted in the US, the others in the UK, Australia and Ireland, suggesting that only limited socio-demographic characteristics are likely to be represented in the sampled population. When this is added to the variability intrinsic to the disorder (symptoms severity, co-morbidity, etc.) it becomes clear that statistical models averaging across data samples are liable to show underlying biases and be poorly generalizable. Conversely, an algorithm that models each individual’s emotional speech reaction has more probability of succeeding in providing a method that is be less encumbered by the disadvantages linked to heterogeneous samples.

If the present findings may upset the traditional way of reasoning, on the other hand, it opens up new clinical and diagnostic scenarios through a change of perspective. The EMF can play a crucial role in diverse diagnostic tools, beyond DD disorder, as a way to extrapolate the hidden traits of a given disorder – bypassing, but embedding, speech. Moreover, the potentiality of EMF spreads over any kind of communicative act (multimodal emotional cues – audio, video, physiological, etc.) and hence, its efficacy can be proved in very diversified contexts.

## Conclusion

In the present work, we explored the possibility of applying a precision approach to the development of a statistical learning algorithm aimed at classifying samples of speech produced by children with developmental disorders (DD) and typically developing (TD) children. Under the assumption that acoustic features of vocal production could not be efficiently used as a direct marker of DD, we propose here a radical change of paradigm. The novel way of reasoning described here opens a promising alternative, by suggesting to apply the Emotional Modulation function (EMF) concept, rather than running analyses on acoustic features *per se*. The EMF is a quantitative way to model the emotional speech reaction of a single individual to a given stimulus. It ‘keeps’ the disorder’s traits while encompassing the natural heterogeneity that individuals manifest during speech production in general. Recognition performance along with comparative results with standard approaches demonstrates the efficacy of the proposed methodology opening up new clinical and diagnostic scenarios through a change of perspective.

## Material and Methods

### Database

In this study, we used the French Child Pathological & Emotional Speech Database (CPESD)^35^ who received the approval by the Ethical Committee of the Pitié-Salpétrière Hospital to conduct recruitment and speech recording of children (as already illustrated in details in^35^). All the thirty-four monolingual participants with communicative verbal skills were recruited in two University departments of child and adolescent psychiatry located in Paris, France. They were diagnosed as AD (Autism Disorders), PDD-NOS (Pervasive Developmental Disorder-Not Otherwise Specified), or SLI (specific language impairment), according to DSM IV criteria^41^. An additional group of 68 TD (Typically Developing) children was recruited in elementary schools. The 102 participants included 21 girls (mean age 11.09; std 4.15) and 81 boys (mean age 9.24; std 2.94). Mean age equally distributed over the three distinct DD diagnoses and the control subjects. Average Verbal Intelligence Score (VIQ) and of Performance Intelligence Quotient (PIQ) resulted 50 (±8.3), 85(±14.4), and 71(11.7) and 77 (±15.3), 76.8(±10.5), 95.4(±14.5) for for AD, PDD-NOS, and SLI subjects, respectively. Further details can be found in^42^.

We will denote as Developmental Disorders (DD) children belonging to categories (AD, PDD-NOS, and SLI). A questionnaire was used to exclude children with learning disorders, a history of speech, language, hearing, or general learning problems. The task administered to the 102 participants was based on a story-telling of a pictured book “Frog where are you?”^36^, wherein a little boy tries to find his frog, escaped during the night. The underlying assumption was that the child is supposed to produce prosodic cues during the story-telling that are correlated to the levels of the emotional valence, which was categorized in three categories by a psychologist: Negative/Neutral/Positive. In total, the pictured book included 15 emotionally negative, six emotionally neutral and five emotionally positive pictures. In the database, nearly 10 hours of recording were collected: 7 h 38 min for TD children, 1 h 35 min for children with AD, 1 h 12 min for children with NOS, and 1 h 56 min for children with SLI. Recordings were then segmented automatically into groups of breaths, using the energy contour. To eliminate sources of perturbation appearing during the recordings (e. g., false-starts, repetitions or environmental noise), the speech segments were further manually processed; only utterances with a complete prosodic contour, i.e., whatever the pronounced words, were kept. For each utterance valence was assessed in three categories by a psychologist: *negative* (labelled as −1), *neutral* (labelled as 0), and *positive* (labelled as +1). Further statistics (number, relative proportion, and mean duration) on those utterances, provided for each valence category, can be found in^35^.

### Speech Analysis

Acoustic features were automatically extracted from the speech waveform on the utterance level using the 2.2 release of the open-source openSMILE feature extractor^43^. Five different feature sets were investigated: large brute-forced feature sets (IS09, IS11, and ComParE), which have all been used for paralinguistic information retrieval, and a smaller, expert knowledge based feature set (eGeMAPS). Those feature sets cover spectral-, source- and duration-related feature space with different levels of detail, cf. Table 1. The first four sets, i.e., IS09, IS11, and ComParE (IS13 in the following), show a clear tendency in enlarging the feature space over the years, by including further low-level acoustic descriptors and associated functionals. Recently, this “brute-forcing” approach has been revisited, with investigations on a small, expert knowledge based feature set, eGeMAPS^44^. A detailed description and implementation of these feature sets is given in^39^. Aggregation of all the available descriptors leads to a feature set of 11227 different descriptors (384 from IS09, 4368 from IS11, 6373 from IS13, and 102 from eGeMAPS). Features redundancy has been solved using mutual correlation analysis. Feature values were then averaged over each utterance hence providing a feature vector for each sentence, leading to a variable number of data for each participant and therein for each affective condition.

### Methods

**Figure 7:**
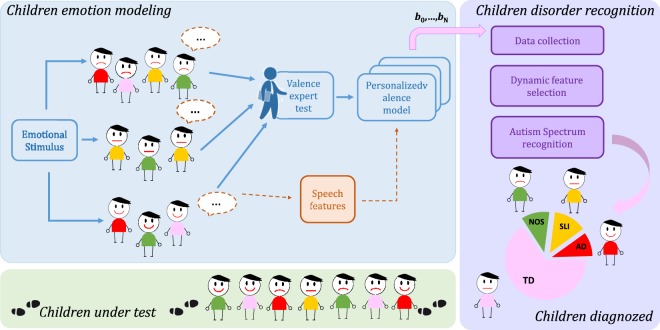
Schematic representation of the whole approach. Scheme of the proposed approach for DD recognition in children using an emotional-valence based speech modelling.

The procedure is summarized by the pictorial scenario shown in Fig. 7. From left to right, we have a group of participants (children), each with a reported diagnosis (red for AD, yellow for SLI, and green for PDD-NOS) and a number of TD subjects (pink dressed) as assessed by experienced psychiatrists. All children were presented with emotional stimuli (here the story-telling, but it can be formulated on different kind of tasks) and their valence attitude was assessed by an expert evaluator during the test administering. The facial expressions in the drawings identify different valence attitude. As observed, there is no a-priori apparent correlation between valence attitude and disorder. Utterances pronounced during the emotional stimulation are recorded (dashed brown arrows) and sent to an automatic speech analysis tool that extracts the acoustic descriptors described in Section 2.1. Descriptors were used to train a personalized valence recognition model. Model coefficients estimated for each participant are collected in a data matrix (data collection block) as the individual signature of emotional attitude in response to a known unique stimulus. The emotional signature of participants with known DD diagnosis are used to train a model, for the automatic discrimination of DD subjects vs control (TD) subjects (pie chart at the bottom-left). Let us consider now in detail each session of the whole methodology.

*N* subjects have been registered collecting a set of speech sequences *N*_si_ i = 1, …, *N*, whose number can be different for each subject. From each sequence, a set of acoustic descriptors are extracted, namely *x*_1_(*k*), …., *x*_Nf_(*k*), where *k* = 1, …, *N*_si_ indicates the sequence and *N*_*f*_ indicates the number of features originally measured. For each subject *i* and for all the relative sequences, we have a feature matrix and an emotion sequence, indicated respectively with ${{\mathbb{X}}}_{{N}_{si}}$ and ${ {\mathcal E} }_{i}$, defined as

1 $${{\mathbb{X}}}_{{N}_{si}}=[\begin{array}{ccc}{x}_{1}(1) & \cdots  & {x}_{{N}_{f}}(1)\\ \vdots  & \ddots  & \vdots \\ {x}_{1}({N}_{si}) & \cdots  & {x}_{{N}_{f}}({N}_{si})\end{array}]\,{ {\mathcal E} }_{i}=[\begin{array}{c}e(1)\\ \vdots \\ e({N}_{si})\end{array}]\,e(k)\in \{-1,0,1\},$$

where label “−1” corresponds to negative valence, label “0” corresponds to neutral valence, and label “+1” corresponds to positive valence. Such labels have been assessed by an expert evaluator.

#### Emotion-related feature selection

The first step aims to select features (i.e., column vector in matrix ${{\mathbb{X}}}_{{N}_{si}}$) that mostly correlate with the emotion vector ${ {\mathcal E} }_{i}$ for each subject *i*.

To do this, we computed the Pearson correlation coefficient *ρ*_*j*,*i*_ between columns of matrix ${{\mathbb{X}}}_{{N}_{si}},{X}_{j}$, $j=1,\ldots ,\,{N}_{f}$, and ${ {\mathcal E} }_{i}$ as follows

2 $${\rho }_{j,i}=\frac{E[({X}_{j}-{\mu }_{{X}_{j}})({ {\mathcal E} }_{i}-{\mu }_{{ {\mathcal E} }_{i}})]}{{\sigma }_{{X}_{j}}\cdot {\sigma }_{{ {\mathcal E} }_{i}}},$$

where *μ*_*Xj*_ and ${\sigma }_{{X}_{j}}\,\,$are the average and the standard deviation of values in column *X*_*j*_, while ${\mu }_{{ {\mathcal E} }_{i}}$ and ${\sigma }_{{ {\mathcal E} }_{i}}$ are the average and the standard deviation of values in vector ${ {\mathcal E} }_{i}$, and *E*[] indicates the expected value. The absolute value of *ρ*_*j*,*i*_ is indicative of the degree of correlation each feature vector *X*_*j*_ has with the corresponding sequence of emotion ${ {\mathcal E} }_{i}$.

Then, for each subject *i*, we finally select those features having an absolute value of *ρ*_*j*,*i*_ larger than 0.7, experimentally set, and achieved the following subset of selected features

3 $${S}_{i}=\{{x}_{j}||{\rho }_{j,i}| > 0.7\},\,j=1,\ldots ,\,{N}_{f},$$

Hence, by the union of the features selected for each subject, we finally obtained a set of selected features for the entire dataset of subjects

4 $$S={\cup }_{i}{S}_{i}.$$

Let us indicate in the following with *N*_*opt*_ the number of selected features, where in general *N*_*opt*_ < *N*_*f*_, and with ${ {\mathcal F} }_{i}=[{f}_{i1},\ldots ,{f}_{i{N}_{opt}}]$ the features for all the subjects. Actually, the set of features are always the same for all the subjects in order to derive an equal number of descriptors for each individual.

#### Personalized Emotional model

 In order to construct a personalized model of emotion for each subject *i*, we preliminary divided the matrix ${ {\mathcal F} }_{i}$ into the three submatrices,$\,{ {\mathcal F} }_{i}{|}_{{ {\mathcal E} }_{i}=-1}\triangleq { {\mathcal F} }_{i}^{-1}$, ${ {\mathcal F} }_{i}{|}_{{ {\mathcal E} }_{i}=0}\triangleq { {\mathcal F} }_{i}^{0}$, and ${ {\mathcal F} }_{i}{|}_{{ {\mathcal E} }_{i}=-1}\triangleq { {\mathcal F} }_{i}^{-1}$ that represent the selected features extracted from the sequences of negative, neutral and positive valence, respectively. Correspondingly, let us denote with ${N}_{si}^{-1}$, ${N}_{si}^{0}$ and ${N}_{si}^{+1}$ the number of sequences for emotions labelled as “−1”, “0” and “+1” respectively, for subject *i* and with ${f}_{ij}^{-1},\,{f}_{ij}^{0}$ and ${f}_{ij}^{+1}$ the feature values of each submatrix, *j* = 1, …, *N*_opt_.

In order to provide a synthetic representation of the emotions picture for a subject, we described the distribution of feature values for each emotion by computing the first, the third and the fourth statistical moments, i.e., the *mean*, the *skewness* and the *kurtosis*, respectively. The skewness parameter is usually used to evidence deviation from Gaussian nature, since it provides a degree of asymmetry of a given distribution of values. The kurtosis instead, also named tailedness, is related to the amount of tails the distribution has with respect to the Gaussian. Both the moments are descriptors of the shape of a distribution more than being descriptors of their localization in the feature space, as conversely the first moment is. Mean *μ*, skewness *sk* and kurtosis *ku* are defined as follows:

5 $${\mu }_{ij}^{-1}=\frac{{\sum }_{k=1}^{{N}_{si}^{-1}}{f}_{ij}^{-1}(k)}{{N}_{si}^{-1}},\,{\mu }_{ij}^{0}=\frac{{\sum }_{k=1}^{{N}_{si}^{0}}\,{f}_{ij}^{0}(k)}{{N}_{si}^{0}},\,{\mu }_{ij}^{+1}=\frac{{\sum }_{k=1}^{{N}_{si}^{+1}}{f}_{ij}^{+1}(k)}{{N}_{si}^{+1}},$$

6 $$s{k}_{ij}^{-1}=\frac{{\sum }_{k=1}^{{N}_{si}^{-1}}{({f}_{ij}^{-1}(k)-{\mu }_{ij}^{-1})}^{3}}{{({\sigma }_{ij}^{-1})}^{3}},\,s{k}_{ij}^{0}=\frac{{\sum }_{k=1}^{{N}_{si}^{0}}{({f}_{ij}^{0}(k)-{\mu }_{ij}^{0})}^{3}}{{({\sigma }_{ij}^{0})}^{3}},\,s{k}_{ij}^{1}=\frac{{\sum }_{k=1}^{{N}_{si}^{+1}}{({f}_{ij}^{+1}(k)-{\mu }_{ij}^{1})}^{3}}{{({\sigma }_{ij}^{1})}^{3}},$$

7 $$\,k{u}_{ij}^{-1}=\frac{{\sum }_{k=1}^{{N}_{si}^{-1}}{({f}_{ij}^{-1}(k)-{\mu }_{ij}^{-1})}^{4}}{{({\sigma }_{ij}^{-1})}^{4}},\,k{u}_{ij}^{0}=\frac{{\sum }_{k=1}^{{N}_{si}^{0}}{({f}_{ij}^{0}(k)-{\mu }_{ij}^{0})}^{4}}{{({\sigma }_{ij}^{0})}^{4}},\,k{u}_{ij}^{1}=\frac{{\sum }_{k=1}^{{N}_{si}^{+1}}{({f}_{ij}^{+1}(k)-{\mu }_{ij}^{1})}^{4}}{{({\sigma }_{ij}^{1})}^{4}},$$

where ${\sigma }_{ij}^{-1},\,{\sigma }_{ij}^{0},\,{\sigma }_{ij}^{1}$ are given by

$${\sigma }_{ij}^{-1}=\frac{{\sum }_{k=1}^{{N}_{si}^{-1}}{({f}_{ij}^{-1}(k)-{\mu }_{ij}^{-1})}^{2}}{{N}_{si}^{-1}},\,{\sigma }_{ij}^{0}=\frac{{\sum }_{k=1}^{{N}_{si}^{0}}{({f}_{ij}^{0}(k)-{\mu }_{ij}^{0})}^{2}}{{N}_{si}^{0}},\,{\mu }_{ij}^{-1}=\frac{{\sum }_{k=1}^{{N}_{si}^{+1}}{({f}_{ij}^{+1}(k)-{\mu }_{ij}^{+1})}^{2}}{{N}_{si}^{+1}}$$

For each subject *i*, a matrix ${{\mathbb{M}}}_{i}$, 3 × 3 *N*_*opt*_ of synthetic descriptors and a corresponding vector of emotions ${{\mathbb{V}}}_{i},\,3\,\times 1$, are built as follows

$${{\mathbb{M}}}_{i}=[\begin{array}{ccc}s{k}_{i1}^{-1} & \cdots  & s{k}_{i{N}_{opt}}^{-1}\,{\mu }_{i1}^{-1}\,\cdots \,{\mu }_{i{N}_{opt}}^{-1}\,k{u}_{i1}^{-1}\,\cdots \,k{u}_{i{N}_{opt}}^{-1}\,\\ s{k}_{i1}^{0} & \cdots \, & s{k}_{i{N}_{opt}}^{0}\,\,{\mu }_{i1}^{0}\,\cdots \,{\mu }_{i{N}_{opt}}^{0}\,k{u}_{i1}^{0}\,\cdots \,k{u}_{i{N}_{opt}}^{0}\\ s{k}_{i1}^{+1} & \cdots  & s{k}_{i{N}_{opt}}^{+1}\,\,{\mu }_{i1}^{+1}\,\cdots \,{\mu }_{i{N}_{opt}}^{+1}\,k{u}_{i1}^{+1}\,\cdots \,k{u}_{i{N}_{opt}}^{+1}\end{array}],\,{\rm{and}}\,{{\mathbb{V}}}_{i}=[\begin{array}{c}-1\\ 0\\ 1\end{array}].$$

The coefficient vector ${ {\mathcal B} }_{i}$ of a multilinear regression estimated by the orthogonal least square approach is then achieved for each subject *i* by

8 $${ {\mathcal B} }_{i}={({{\mathbb{M}}}_{i}^{T}{{\mathbb{M}}}_{i})}^{-1}{{\mathbb{M}}}_{i}^{T}{{\mathbb{V}}}_{i}.$$

Coefficient vector ${ {\mathcal B} }_{i}$ represents the personalized model of emotion of each subject *i*, his/her EMF, i.e., the way the subject reacts to specific emotional stimuli provided during the task with his/her own speech frequency alteration. The assumption is that EMF coefficients ${ {\mathcal B} }_{i}$ may be used to discriminate TD subjects from DD subjects, by concealing the different emotional picture of the two groups of subjects.

#### An emotional-guided diagnostic tool for DD patients

By collecting as rows the coefficient vector ${ {\mathcal B} }_{i}$ for all the subjects – for simplicity sorted according to the disorder (i.e., control, label 1, label 2 and label 3) – we constructed a data matrix ${\mathbb{D}}$, a corresponding binary disorder-labelled vector, ${\mathbb{Y}}$ and a 4-classes disorder-labelled vector, ${\mathbb{L}}$, as follows

9 $${\mathbb{D}}=[\begin{array}{c}\mbox{--}{ {\mathcal B} }_{1}\mbox{--}\\ \vdots \\ \mbox{--}{ {\mathcal B} }_{i}\mbox{--}\\ \vdots \\ \mbox{--}{ {\mathcal B} }_{N}\mbox{--}\end{array}],\,{\mathbb{Y}}=[\begin{array}{c}0\\ \vdots \\ 0\\ 1\\ \vdots \\ 1\end{array}],\,{\mathbb{L}}=[\begin{array}{c}0\\ \vdots \\ 0\\ 1\\ \vdots \\ 2\\ \vdots \\ 3\end{array}]$$

where values ${\mathbb{Y}}\equiv 1$ correspond to any ${\mathbb{L}} > 0$. Due to the low number of cases for each disorder, 10 (NOS), 13 (SLI) and 11 (AD) respectively, we decided to develop a binary classification model able to recognize TD subject from DD subject. Hence, we considered the labelled vector ${\mathbb{Y}}$ as ground truth.

Under the assumption that features selected play a crucial role in the recognition performance, especially due to the heterogeneity of the test set, we applied here a dynamic feature selection (DFS) procedure intended to optimally select model features according to each specific test data. More specifically, in line with the recently developed methodology^37,38^, we design the following three-level DFS approach:

Test-independent feature elimination step: Starting from the training set, the Fisher Discriminant Score (FDS) is used to sort all the available features according to their compliance with the classification problem. In particular, a feature to be included requires that at least one class-distribution is statistically different from the others. Let us consider a two-class problem, and let us assume that each class has *L*_d_, d = {1, 2} training vectors ${ {\mathcal B} }_{i}$ each formed by elements *b*_*ik*_, *i* = 1, .., *L*_d_, k = 1, …, *M*, with *M* as the total number of features. Then, for each feature *k*, *FDS*_*k*_ is defined as the ratio between intra-class and inter-class variance and it is estimated as follows:

10 $$FD{S}_{k}=S{B}_{k}/S{W}_{k},$$

where *SB*_*k*_ is the intra-class and *SW*_*k*_ is the inter-class variance. In particular, *SB*_*k*_ is defined as

11 $$S{B}_{k}={\sum }_{d=1}^{2}{({b}_{\{Y={Y}_{d}\}k}-{\bar{b}}_{k})}^{2},$$

with *Y*_*d*_ = {0, 1}, ${\bar{b}}_{k}$ is the average of the feature values *b*_*k*_ computed over all the classes. For each feature, *k*, *SB*_*k*_ quantifies the sum of dispersions of training samples in a class (by their variance) with respect to the global average value of that feature.

On the other hand, for each feature *k*, we also computed

12 $$S{W}_{k}={\sum }_{d=1}^{2}\frac{1}{{L}_{d}}{\sum }_{i=1}^{{L}_{d}}{({b}_{{\{Y={Y}_{d}\}}_{i}k}-{\bar{b}}_{dk})}^{2},$$

with ${b}_{{\{Y={Y}_{d}\}}_{i}k}$ as the *i-th* element of feature *k-th* for the class labelled as *Y*_*d*_ and ${\bar{b}}_{dk}$ is the average value of feature *k-th* in the class labelled as *Y*_*d*_. For each feature, *SW*_*k*_ quantifies the dispersion of elements in a class *d*, with respect to their average value, i.e., the inter-class variance.

Higher values for *FDS*_*k*_ indicate that the feature *k* is representative of at least one class and hence will be maintained. For this task, we will define a threshold value *th*_FDS_ and define the Condition 1 (C_1_) as follows

13 $${C}_{1}:{b}_{k}{\rm{will}}\,{\rm{be}}\,{\rm{kept}}\,{\rm{iff}}\,\{{FD}{{S}}_{{k}} > {t}{{h}}_{{FDS}}\},$$

Online test-dependent feature elimination step: This step utilizes two criteria for selecting a temporary subset of features. The decision is made in accordance with the sample reservoir containing the information about the class distributions and the test sample newly acquired. This step is intended to online remove the features in which either the test sample is far from all class distributions (feature outlier values) or it is surrounded by samples of different classes (high probability of misclassification). In order to achieve these targets, the algorithm selects a feature only if it fulfils the two following criteria. Let us denote with *s* the test sample and with *s*_*k*_ its k-th element, for brevity test element.

The *first* criterion is the ratio between the Mahalanobis distances of the test element *s*_*k*_ from the two class distributions, hereinafter denoted as *MR*_k_ (*s*_*k*_). This value is calculated for each feature *k* as follows:

14 $$M{R}_{k}({s}_{k})={M}_{k}({s}_{k},Y={Y}_{1})\cdot \frac{{M}_{k}(s,Y={Y}_{1})}{{M}_{k}(s,Y={Y}_{2})},$$

where ${M}_{k}({s}_{k},Y={Y}_{d})$ is the Mahalanobis distance of the test element *s*_*k*_ from the training samples belonging to the class labelled as *Y*_*d*_ and it is defined as

15 $${M}_{k}({s}_{k},Y={Y}_{d})=({s}_{k}-{\bar{b}}_{dk})\ast {({Cov}({b}_{\{Y={Y}_{d}\}k}))}^{-1}\ast {({s}_{k}-{\bar{b}}_{dk})}^{{\rm{T}}},$$

where $\,Cov({b}_{\{Y={Y}_{d}\}k})$ is the covariance of the feature matrix of samples belonging to the class labelled as *Y*_*d*_. The descriptor $M{R}_{k}({s}_{k})$ provides a quantitative measure of the distance of the test sample from the two classes. Lower values of $M{R}_{k}({s}_{k})$ indicate that the test element *s*_*k*_ is close to a given class while being far from the other class. Hence, defined a threshold value *th*_MR_, a Condition 2 (C_2_) will be formulated as follows:

16 $${C}_{2}:{b}_{k}{\rm{will}}\,{\rm{be}}\,{\rm{kept}}\,{\rm{iff}}\,\{M{R}_{k} < t{h}_{MR}\}.$$

The *second* criterion evaluates the maximum probability for the test element *s*_*k*_ to belong to each class distribution. For the k-th feature, *P*_k_ is computed as follows:

17 $${P}_{k}({s}_{k})={\max }_{d}(\frac{1}{\sqrt{2\pi }{\sigma }_{dk}}\exp (-\frac{{({s}_{k}-{\bar{b}}_{dk})}^{2}}{2{\sigma }_{dk}^{2}})),$$

where ${\sigma }_{dk}$ is the standard deviation of training sample values for feature k-th in the class labelled as *Y*_*d*_. Higher values for ${P}_{k}({s}_{k})$ indicate that the test element *s*_*k*_ has a high probability to be correctly represented by a given class distribution. For this reason, defined a threshold value *th*_P _, a Condition 3 (C_3_) will be formulated as follows:

18 $${C}_{3}:{b}_{k}{\rm{will}}\,{\rm{be}}\,{\rm{kept}}\,{\rm{iff}}\,\{{P}_{k} > t{h}_{P}\}$$

For each test element *s*_*k*_, only the features *b*_k_ in the training set that respect conditions C_1_ and in cascade conditions C_2_–C_3_ are used to construct the predictive model. In our approach, a Support Vector Machine (SVM) with linear kernel and standard parameters setting was preferred for the scope. Features are selected at each test step; features eliminated in a step will be re-inserted in the training set and re-considered for the feature selection procedure at the next step in presence of a different test data.
